# Impact of coronavirus disease 2019 pandemic on good clinical practice trials in oncology

**DOI:** 10.37349/etat.2023.00183

**Published:** 2023-10-30

**Authors:** Veronica Agostinelli, Zelmira Ballatore, Giulia Ricci, Alessandra Lucarelli, Michela Burattini, Lorenzo Mariotti, Claudia Catani, Valentina Tarantino, Rossana Berardi

**Affiliations:** Cancer Treatment Centers of America Medicine & Science, USA; ^1^Department of Medical Oncology, Università Politecnica delle Marche, 60126 Ancona, Italy; ^2^Department of Medical Oncology, Azienda Ospedaliero-Universitaria delle Marche, 60126 Ancona, Italy

**Keywords:** Coronavirus disease 2019, pandemic, cancer patients, oncology, clinical trials, good clinical practice

## Abstract

**Aim:**

Coronavirus disease 2019 (COVID-19) became pandemic on 11th March 2020 and it deeply stressed the healthcare system. Cancer patients represent a vulnerable population, so many recommendations have been approved to ensure optimal management. Clinical research was notably impacted by COVID too. This review aims to analyze the challenges occurred during a pandemic for the management of enrolled patients (enrollment, use of telemedicine visits, study procedures) and for the clinical trials system (from feasibility to selection visit, site initiation visit, monitorings, use of e-signature, deviations and discontinuations).

**Methods:**

The studies included in the present review were selected from PubMed/Google Scholar/ScienceDirect databases.

**Results:**

During the first phase of pandemic many clinical trials were suspended in accrual and, as the pandemic progressed, recommendations were established to guarantee the safety and the continuity of care of enrolled patients. In addition, lot of new strategies was found during the pandemic to reduce the negative consequences on clinical trial performance and to guarantee new opportunities of care in the respect of good clinical practice (GCP) in a bad scenario.

**Conclusions:**

Among all modifiers, investigators would prefer to maintain the positive ones such as pragmatic and simplified trial designs and protocols, reducing in-person visits when not necessary and to minimizing sponsor and contract research organizations (CROs) visits.

## Introduction

On 30th January 2020 the World Health Organization (WHO) declared coronavirus disease 2019 (COVID-19) a public health emergency and on 11th March 2020 the pandemic [1]. Because of COVID-19 airborne transmission, the only effective measures to prevent COVID-19 community spread were social distance, containment, testing and isolating cases [2].

During the first phases of the pandemic, guidelines were established to limit the virus spread [3] and hospitals had to save personal protection equipment and resources in order to manage COVID-19 patients [4]. Pandemic deeply stressed the healthcare systems trying to preserve both patients and healthcare workers [5, 6].

Above all, cancer patients are a vulnerable population due to the immunosuppressed status caused by chemotherapy, radiotherapy or surgery and the consequent increased risk of infections [7]. Many COVID-19 guidelines have been released in the last two years to manage cancer patients, to reorganize healthcare services and professionals to fight and prevent spread of disease [7].

Despite all of the above, the virus spread so widely within hospitals and patients experienced anxiety and fear of contagious disease in a place that should have been considered safe and healthy [8].

Therefore, to reduce the risk of COVID-19 infection in cancer patients, oncologists tried to reduce hospital visits, postponing appointments and delaying oncological treatment when applicable [9]. In addition to this, hospitalizations due to severe acute respiratory syndrome coronavirus 2 (SARS-CoV-2) infection adversely affected the prognosis of cancer disease [7].

Among cancer care, clinical trials were notably affected by COVID-19, too. Despite the fact that clinical trials represent an important tool in cancer care, institutions worldwide have decided to suspend clinical trials recruitment at the beginning of the pandemic [10]. The guarantee of patient safety and of data integrity were the keys consideration in deciding whether to interrupt or continue clinical trials.

The present review aims to analyze the impact of the COVID-19 pandemic on clinical trials and to identify strategies to guarantee new opportunities of care in the respect of good clinical practice (GCP) in a bad scenario.

## Materials and methods

The studies included in the present review were selected from PubMed/Google Scholar/ScienceDirect databases. We used the terms “COVID-19”, “Pandemic”, “Clinical Trial”, “GCP”, “Oncology”, and “Guidelines” to select papers focusing on the impact of COVID-19 in oncological clinical trials.

Article search was conducted independently by the authors. The following inclusion criteria were used to identify the studies:

(1). Manuscripts on clinical trial including original research, retrospective studies, observational studies, review articles, surveys, and guidelines.
(2). Target population: cancer patients and patients enrolled in oncological clinical trials.

In addition, the search engine clinicaltrial.gov was used to access clinical trial data.

The considered time was the pandemic starting from the 30th of January 2020 that is when the WHO proclaimed COVID-19 a public health emergency.

Exclusion criteria were:

(1). Meeting or conference abstract only.
(2). Duplicate papers.
(3). Articles not written in English language.

**Figure 1:**
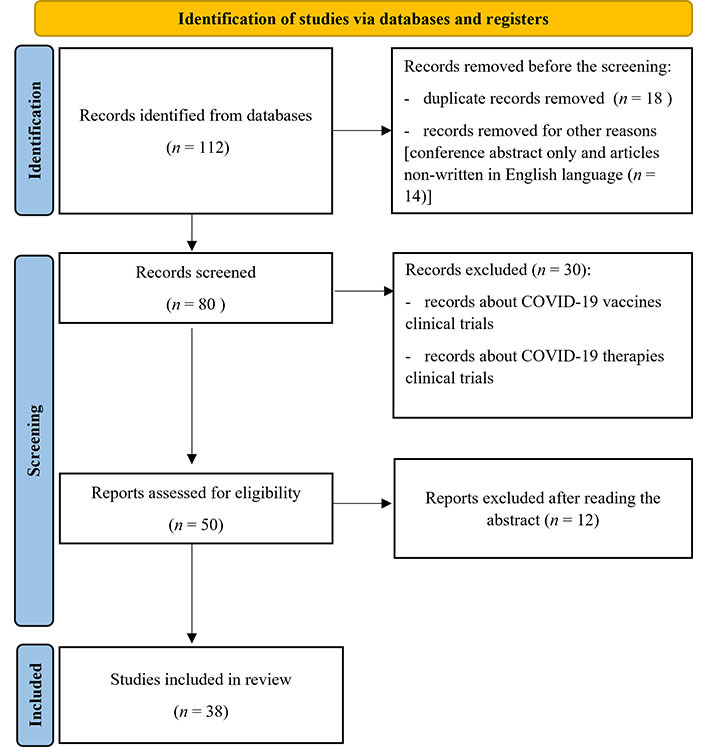
Flowchart of research screening

The flowchart of this study is shown in Figure 1.

## Results

### The impact of COVID-19 on clinical trials in oncology

Six days after WHO declared COVID-19 a pandemic, the National Institutes of Health (NIH) issued a guide for the management of clinical trials focusing on “safety and welfare of human subject participants and research staff” [11].

Consequently, the Food and Drug Administration (FDA) developed recommendations to assist sponsors in ensuring safety and compliance with GCP and avoiding risks to trial data integrity [12]. In March 2020, FDA published guidelines for clinical trials management which were updated several times during pandemic [13]. The paper, in particular, aided industries, investigators, and institutional review boards in ensuring the safety of human subjects, maintaining compliance with GCP, and reducing risks to data integrity [12].

In the same period other regulatory agencies such as the European Medicines Agency (EMA) [14] and Italian Agency of the Drugs [Agenzia Italiana del Farmaco (AIFA)] [15] published guidelines to regulate the management of clinical trials.

To reduce patients’ travel during the early pandemic phase some institutions have suspended recruitment [4], while other trials experienced important adjustments [16].

According to ClinicalTrials.gov, 1,052 trials were suspended from March to April 2020 and 905 of them were suspended due to the pandemic Tables 1 and 2 [17].

**Table 1:** Suspended, active non-recruiting and recruiting oncological clinical trials throughout the World (W), the United States (U) and Italy (I) from the beginning of pandemic to the first six months of 2022

**Study status**	**30/01/2020 to 30/07/2020**			**01/08/2020 to 31/12/2020**			**01/01/2021 to 30/06/2021**			**01/07/2021 to 31/12/2021**			**01/01/2022 to 30/06/2022**		
	**W**	**U**	**I**	**W**	**U**	**I**	**W**	**U**	**I**	**W**	**U**	**I**	**W**	**U**	**I**
Suspended	28	18	1	14	11	/	20	17	/	16	14	/	16	11	/
Active, non-recruiting	302	155	37	259	138	28	250	116	30	162	69	18	92	39	3
Recruiting	1,421	577	100	1,723	703	117	2,511	945	169	2,401	960	143	2,362	932	113
Total	3,364	1,160	220	3,394	1,220	225	4,335	1,439	291	3,959	1,407	228	4,006	1,290	208

The 30/01/2020 means January 30th, 2020; other dates are in the same format. /: not applicable

**Table 2:** Suspended, active non-recruiting and recruiting oncological phase 1, 2, 3 clinical trials respectively throughout the world from the beginning of pandemic to the first six months of 2022 (30/01/2020 to 30/06/2022)

**Study status**	**Phase 1**	**Phase 2**	**Phase 3**
Suspended	33	41	5
Active, non-recruiting	236	357	118
Recruiting	2,368	3,474	818
Total	3,592	5,466	1,464

In the first phase of the pandemic, there was a 41.8% reduction in recruiting cancer patients in clinical trials compared to the same month of the previous year [18].

According to the IQVIA (a human data science company) survey, between 23 March and 3 April 2020 clinical trials enrollment was considerably affected, particularly in the United States and in Europe, with 20% and 14% of all institutions continuing to enroll patients, respectively in each country.

The pandemic had a “moderate” or “high” impact on patient visits too (delayed or canceled), according to over 60% of investigators. The majority of responders (80%) expected protocol deviations due to incomplete patient visit data [19].

To maintain social distance, delays or omitted laboratory or instrumental tests, postponed visits and ways other than the specified study schedule of evaluation were allowed, while telemedicine has increasingly taken over [20].

### Risk mitigation strategies in clinical trial: focus on the patient

As a result of the hospital access limitation, there were issues in controlling studies procedures and activities, from follow-up visits to the imaging and laboratory testing, causing delays in data collection and integrity [21]. Patients reported the interest in remaining on trial, but they also wanted to maintain adequate social distance to reduce the risk of infection [22]. The most significant paradox is associated to the virus’s spread in hospitals, the place which is the heart of healthcare and where patients are treated and need to feel protected. During the pandemic patients were terrified to go to the hospital for fear of contracting the virus [23].

In order to reduce the contagious risk and to ensure the adherence to GCP, telemedicine and remote visits have been implemented as important tools for patient’s continuum of care [24].

Institutions permitted to reduce the frequency of follow up in-person visits and to employ telemedicine, including virtual visits and questionnaire administration through emails or phone [4].

It was allowed for study blood tests to be performed in local laboratories when patients resided distant from the site as long as safety and scheduling protocols were followed, reducing the discontinuations and/or protocol deviations [4–25].

During the pandemic, radiologists were primarily focused to perform chest computed tomography (CT) scans in COVID-19 patients and this caused delays in both cancer staging and planned assessment [26].

Difficulties and concerns occurred throughout the COVID-19 pandemic are highlighted in Table 3.

**Table 3:** Difficulties and concerns during COVID-19 pandemic

**Difficulties**	**Concerns**
Incomplete patient visit data and protocol violations due to delayed or deleted visits	Loss of compliance with GCP
Radiologists mainly dedicated to performing chest CT scan in COVID-19 patients	Avoid infection for both cancer patients and healthcare professionals
	Several data lost
	The continuum of care could not be guaranteed

### Risk mitigation strategies in clinical trial: focus on the system

Given the hospital’s limited access, the sponsors and contract research organizations (CROs) carried out remote site initiation visits (SIV) and monitoring visits [27].

It was possible to enhance technology, by progressively phasing out obsolete tools (such as fax) and ensuring the usage of better systems especially with the increase of smart-working [24, 28, 29].

Remote monitoring enabled saving money including travel costs, but also healthcare professionals to save time by reducing unnecessary and non-trial relevant material [25]. In fact, investigators are often expected to capture insignificant occurrences only to acknowledge them, resulting in an excess of paperwork, requests for revisions to patients' notes, and time spent with poor clinical significance [25].

The usage of e-signature for informed consent and other study documents has also grown in monitoring and telemedicine [27]. Strategies of risk mitigation are summarized in Table 4.

**Table 4:** Changes and modifications during COVID-19 pandemic

**Type of risk mitigation strategies**	**Change and modifications**
Risk mitigation strategies: focus on the patients	Improvement of telemedicine to reduce in-person visits
	Blood samples in local laboratories
	Delay imaging studies or allow it in qualified local radiology
Risk mitigation strategies: focus on the system	Improvement of smart-working
	Remote visit and monitoring
	Improvement use of e-signature

### Consequences of COVID-19 in clinical trials

COVID-19 had a significant influence on clinical trials, both in terms of patient recruitment and research in general, with short and long-term consequences [30].

At the beginning of the pandemic, regulatory agencies suspended screening and patient recruitment in clinical trials as a precautionary measure. As a result, many new important opportunities of care were lost [31].

As the world has adjusted to the pandemic and the global lockdown has been lifted, clinical studies have resumed. The same guidelines that regulated the suspension and/or continuation of clinical trials were used for their reinstatement [32].

During the second phase of the pandemic, however, recruitment and clinical trial management had to be restarted. It was important to approve improvements to streamline and simplify procedures, to expand and make the scheduling more flexible and to minimize the requirements for data collection [27].

All the modifications caused by pandemic have encouraged study sponsors, CROs, research programs, and regulatory authorities to recognize protocol modifications and deviations as an unavoidable issue, emphasizing the possibility of conducting trials in a more efficient manner [27].

## Discussion

In the last 10 years, clinical research of new cancer drugs plus better surgery techniques, radiotherapy and supportive care have all contributed to cancer patients’ increased survival [33].

The COVID-19 pandemic had an important impact on clinical research and on the development of new drugs especially during the first phase of a pandemic when recruiting was halted. When considering the pandemic scenario, two issues arise: is there any difference between cancer patients in clinical trials and standard patients? How long a clinical trial enrollment may be postponed?

Many literature data demonstrated that clinical trial patients have better outcomes for the superior clinical procedures [31]. Moreover, in order to be enrolled and satisfy all inclusion criteria (without exclusion ones), patients must have ideal clinical conditions. As a result of postponing enrollment, patients may worsen their conditions, become ineligible for the trial and lose the new drug opportunity [34]. Furthermore, sometimes enrollment in clinical trials may be the last chance for patients if there is no more therapy available [31]. With suboptimal treatments, cancer patients might have an increased risk of death regardless COVID-19 disease [34].

However, clinical trials are often available only in select accredited cancer centers and need more frequent in-person visits than standard treatments. During the pandemic, to limit travel and virus spread, sites began to guarantee, when available, virtual visits according to the new regulatory agency’s guidelines [35].

Since the beginning of the pandemic the use of telemedicine has increased more and more and its use continues even in a better pandemic condition. Telemedicine could be used for remote visits, but also to manage patients’ symptoms and adverse events [36]. Furthermore, telemedicine allows patients to not travel to clinical trial sites decreasing expenses and absences from work: this may contribute to an increase in participation in clinical trials [33]. On the other hand, telemedicine leads to a loss in a physician-patient relationship. In-person visits provide with greater privacy, improved communication between oncologists and patients and the opportunity to empathize, resulting in a trusting relationship and elderly patients may lack the skill to use technology and an internet connection [37, 38].

Before the pandemic, blood samples could not be tested outside of the trial site or in affiliated and certified laboratories. During the COVID-19 pandemic the regulatory agencies were more flexible and agreed on procedures to facilitate both patients and physicians allowing the use of unaffiliated laboratories [12].

Imaging studies endured modifications too. Clinical trials required high imaging’ frequency to evaluate the disease status in a timely manner [33]. During the pandemic, it was frequently necessary to delete or to delay imaging due to the radiologists or the patientsʼ difficulties: regulatory agencies and sponsors did not consider these events protocols’ deviations. Delay imaging could represent a significant improvement for patients in the future, decreasing their exposure to radiation and iodinated contrast. Thus, the usefulness of such close imaging might be evaluated against the use of possibly non-surrogate endpoints without compromising clinical trial data and results [33].

Research system required to be modified and re-adjust to guarantee the re-start or continuations of clinical trials [33]. Through the use of remote site visits and monitoring, the approach to trials has been significantly changed and simplified, saving both investigators and administration time and consequently improving the performance of clinical trials units and the quality of patient care [25].

It seems that the pandemic impact on clinical research will cause an increase in cancer deaths as a result of missed screening, delayed diagnosis and reduced oncology care.

In addition, significant implications for future outcomes and drug development will be apparent due to the reduction of enrollment in clinical trials in the first phase of the pandemic. Nevertheless, the pandemic offered an opportunity for clinical trial innovation, emphasizing aspects that could be simplified, modernized, and adapted in order to maintain research quality and integrity [37, 38].

Longer follow-up will be required in the future to analyze the post-pandemic impacts on clinical studies, including objectives, and outcome measures.

In conclusion, the COVID-19 pandemic changed many aspects of oncology care and will leave long-term consequences especially for clinical research. Among all modifiers, investigators would prefer to maintain the positive ones such as pragmatic and simplified trial designs and protocols, reducing in-person visits when not necessary and to minimizing sponsor and CROs visits. All of these elements would enhance clinical trials management. The pandemic provides an opportunity to focus on what is required, effective, and relevant for cancer patients, healthcare workers, and cancer drug development.

## Abbreviations

COVID-19: coronavirus disease 2019
CROs: contract research organizations
GCP: good clinical practice
WHO: World Health Organization

## Declarations

### Copyright

© The Author(s) 2023.

## References

[1] Coronavirus disease (COVID-19) pandemic [Internet]. https://www.who.int/emergencies/diseases/novel-coronavirus-2019/.

[2] COVID-19 overview and infection prevention and control priorities in non-U.S. healthcare settings [Internet]. https://www.cdc.gov/coronavirus/2019-ncov/hcp/non-us-settings/overview/index.html/.

[3] North CM, Dougan ML, Sacks CA (2020). Improving clinical trial enrollment—in the Covid-19 era and beyond. N Engl J Med.

[4] Ndumele A, Park KU (2021). The impact of COVID-19 on national clinical trials network breast cancer trials. Curr Breast Cancer Rep.

[5] Ballatore Z, Merloni F, Ranallo N, Bastianelli L, Vitarelli F, Cantini L (2022). Cancer patient perspective in the arena of COVID-19 pandemic. Psychooncology.

[6] de Joode K, Dumoulin DW, Engelen V, Bloemendal HJ, Verheij M, van Laarhoven HWM (2020). Impact of the coronavirus disease 2019 pandemic on cancer treatment: the patients’ perspective. Eur J Cancer.

[7] Al-Quteimat OM, Amer AM (2020). The impact of the COVID-19 pandemic on cancer patients. Am J Clin Oncol.

[8] Apisarnthanarak A, Siripraparat C, Apisarnthanarak P, Ullman M, Saengaram P, Leeprechanon N (2021). Patients’ anxiety, fear, and panic related to coronavirus disease 2019 (COVID-19) and confidence in hospital infection control policy in outpatient departments: a survey from four Thai hospitals. Infect Control Hosp Epidemiol.

[9] Dai M, Liu D, Liu M, Zhou F, Li G, Chen Z (2020). Patients with cancer appear more vulnerable to SARS-CoV-2: a multicenter study during the COVID-19 outbreak. Cancer Discov.

[10] Unger JM, Cook E, Tai E, Bleyer A (2016). The role of clinical trial participation in cancer research: barriers, evidence, and strategies. Am Soc Clin Oncol Educ Book.

[11] Guidance for NIH-funded clinical trials and human subjects studies affected by COVID-19 [Internet]. https://nexus.od.nih.gov/all/2020/03/17/guidance-for-nih-funded-clinical-trials-and-human-subjects-studies-affected-by-covid-19/.

[12] Conduct of clinical trials of medical products during the COVID-19 public health emergency: guidance for industry, investigators, and institutional review boards [Internet]. https://collections.nlm.nih.gov/catalog/nlm:nlmuid-9918351277706676-pdf/.

[13] van Dorn A (2020). COVID-19 and readjusting clinical trials. Lancet.

[14] Guidance to sponsors on how to manage clinical trials during the COVID-19 pandemic [Internet]. https://www.ema.europa.eu/en/news/guidance-sponsors-how-manage-clinical-trials-during-covid-19-pandemic.

[15] Clinical trials’ management in Italy during the COVID-19 (*coronavirus disease 19*) emergency [Internet]. https://www.aifa.gov.it/documents/20142/871583/Comunicato_gestione_studi_clinici_in_emergenza_COVID-19_EN_12.03.2020.pdf.

[16] Unger JM, Blanke CD, LeBlanc M, Hershman DL (2020). Association of the coronavirus disease 2019 (COVID-19) outbreak with enrollment in cancer clinical trials. JAMA Netw Open..

[17] NCTN group chairs: cancer trials take backseat to clinical care amid COVID-19 pandemic [Internet]. https://cancerletter.com/articles/20200410_1/.

[18] Upadhaya S, Yu JX, Oliva C, Hooton M, Hodge J, Hubbard-Lucey VM (2020). Impact of COVID-19 on oncology clinical trials. Nat Rev Drug Discov.

[19] Patil R, Varner C Delivering clinical trial continuity during COVID-19.

[20] Kuderer NM, Choueiri TK, Shah DP, Shyr Y, Rubinstein SM, Rivera DR, COVID-19 and Cancer Consortium (2020). Clinical impact of COVID-19 on patients with cancer (CCC19): a cohort study. Lancet.

[21] ClinicalTrials.gov [Internet]. https://clinicaltrials.gov/ct2/search/advanced?cond=&term=&cntry=&state=&city=&dist=.

[22] Agostinelli V, De Filippis C, Torniai M, Rocchi MBL, Pagliacci A, Ricci G (2023). Primum non nocere: how to ensure continuity of care and prevent cancer patients from being overlooked during the COVID-19 pandemic. Cancer Med.

[23] Hall E, Lewis R, Snowdon C (2020). Life after COVID-19 for cancer clinical trials. Int J Radiat Oncol Biol Phys.

[24] Fontana E, Arkenau HT (2020). Oncology clinical trials during the COVID-19 outbreak: lessons learnt during the crisis and future opportunities. Cancer Treat Rev.

[25] Caldarella C, Cocciolillo F, Taralli S, Lorusso M, Scolozzi V, Pizzuto DA (2022). The impact of the COVID-19 pandemic on oncological disease extent at FDG PET/CT staging: the ONCOVIPET study. Eur J Nucl Med Mol Imaging.

[26] Waterhouse DM, Harvey RD, Hurley P, Levit LA, Kim ES, Klepin HD (2020). Early impact of COVID-19 on the conduct of oncology clinical trials and long-term opportunities for transformation: findings from an American Society of Clinical Oncology Survey. JCO Oncol Pract.

[27] Tolaney SM, Lydon CA, Li T, Dai J, Standring A, Legor KA (2021). The impact of COVID-19 on clinical trial execution at the Dana-Farber cancer institute. J Natl Cancer Inst.

[28] Perez-Gracia JL, Awada A, Calvo E, Amaral T, Arkenau HT, Gruenwald V (2020). ESMO Clinical Research Observatory (ECRO): improving the efficiency of clinical research through rationalisation of bureaucracy. ESMO Open.

[29] Gregucci F, Caliandro M, Surgo A, Carbonara R, Bonaparte I, Fiorentino A (2020). Cancer patients in Covid-19 era: swimming against the tide. Radiother Oncol.

[30] Massari F, Mollica V, Salvagni S, Tognetto M, Ardizzoni A (2020). Oncology clinical trials in the time of COVID-19: how a pandemic can revolutionize patients’ care. Future Oncol.

[31] Asaad M, Habibullah NK, Butler CE (2020). The impact of COVID-19 on clinical trials. Ann Surg.

[32] Nabhan C, Choueiri TK, Mato AR (2020). Rethinking clinical trials reform during the COVID-19 pandemic. JAMA Oncol.

[33] Tella SH, Kommalapati A, Alberts SR, McWilliams R, Mahipal A (2020). Collateral damage of COVID-19 pandemic on oncology clinical trials. Chin Clin Oncol.

[34] Wosik J, Fudim M, Cameron B, Gellad ZF, Cho A, Phinney D (2020). Telehealth transformation: COVID-19 and the rise of virtual care. J Am Med Inform Assoc.

[35] Mooney M, McCaskill-Stevens W Interim guidance for patients on clinical trials supported by the NCI cancer therapy evaluation program and the NCI community oncology research program; 2020.

[36] Gensheimer MF, Yom SS, Soto N, Dignam JJ, Le QT, Machtay M (2020). Multicenter clinical cancer research after COVID-19: a perspective from NRG oncology. Int J Radiat Oncol Biol Phys.

[37] de Las Heras B, Saini KS, Boyle F, Ades F, de Azambuja E, Bozovic-Spasojevic I (2020). Cancer treatment and research during the COVID-19 pandemic: experience of the first 6 months. Oncol Ther.

[38] Sharpless NE (2020). COVID-19 and cancer. Science.

